# Protocol for the genome-wide identification of intrinsic transcription factor binding motifs by mammalian-optimized pull-down sequencing

**DOI:** 10.1016/j.xpro.2026.104513

**Published:** 2026-04-21

**Authors:** Xuan Jiang, Wenxiang Zhang

**Affiliations:** 1 Shanghai Institute of Immunology, Department of Immunology and Microbiology, Shanghai Jiao Tong University School of Medicine, Shanghai 200025, China

**Keywords:** ChIPseq, Immunology, Molecular Biology

## Abstract

Chromatin immunoprecipitation sequencing (ChIP-seq) maps *in vivo* transcription factor (TF) occupancy under native chromatin conditions. Here, we present a protocol for genome-wide profiling of intrinsic TF motifs using protein-DNA pull-down sequencing (PD-seq), a complementary *in vitro* technique based on DNA affinity purification sequencing (DAP-seq) with optimizations for mammalian systems. We describe steps for TF purification, naked genomic DNA preparation, protein-DNA pull-down, and sequencing-based analysis. Applied to FOXP3, PD-seq identifies T_n_G microsatellite repeats as a preferred motif. The protocol bypasses antibody dependence and applies to any affinity-tagged DNA-binding protein.

For complete details on the use and execution of this protocol, please refer to Zhang et al.^1^

## Highlights

• Instructions for recombinant TF expression and affinity purification
• Steps for preparing fragmented naked genomic DNA for pull-down
• Guidance on protein-DNA pull-down and sequencing library construction
• Procedures for bioinformatic analysis and *de novo* motif discovery

## Abstract (editor highlights)

Publisher’s note: Undertaking any experimental protocol requires adherence to local institutional guidelines for laboratory safety and ethics.

## Graphical abstract

**Figure:**
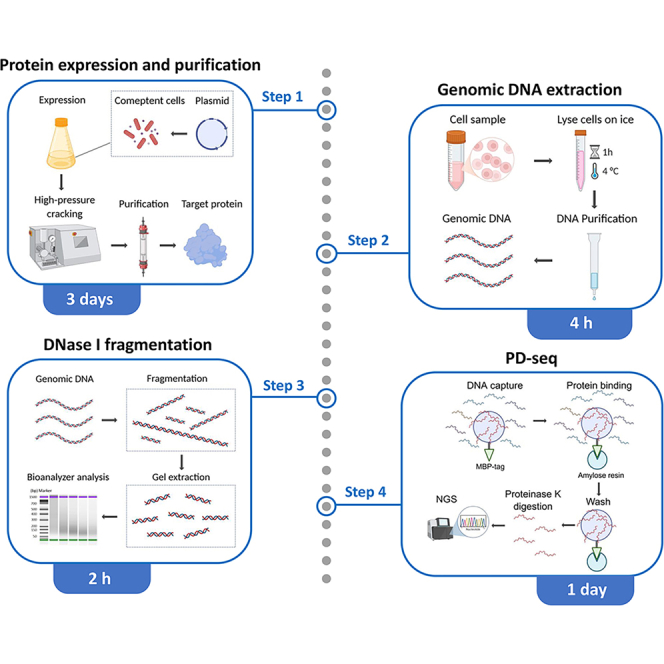

## Before you begin

ChIP-seq is the gold standard for mapping TF (transcription factor) occupancy in vivo,^2^^,^^3^^,^^4^ providing critical insights into TF binding under native chromatin conditions. While ChIP-seq captures the influence of chromatin environment and co-factors, PD-seq is designed as a complementary in vitro approach to dissect the intrinsic DNA-binding specificity of a TF in a purified system. Based on the principle of DAP-seq^5^ but with key optimizations for mammalian systems, PD-seq uses fragmented naked genomic DNA and recombinant protein to directly interrogate the sequence preferences of a TF independent of cellular context, offering a controlled system to complement in vivo occupancy data.

### Innovation

Unlike DAP-seq, which has been primarily applied to plant TFs,^6^ PD-seq incorporates optimizations in protein purification and experimental workflow tailored to the challenges of mammalian studies, enabling the discovery of novel binding modalities such as microsatellite repeats. In contrast to methods like GHT-SELEX^7^ that involve multiple rounds of selection and amplification, PD-seq employs a single-round pull-down strategy directly from complex genomic DNA. This design difference reflects distinct methodological approaches: GHT-SELEX enriches for high-affinity binders through iterative selection, whereas PD-seq captures binding events in a single step without intervening amplification, offering a complementary approach for interrogating intrinsic TF specificity.

### Protein selection and cloning considerations

1. Employ an N- or C-terminal affinity tag such as MBP, His-MBP, or other well-characterized tags compatible with high-affinity purification resins.^8^
2. Although FOXP3 (ΔN) is presented as a model TF throughout this protocol, it may be substituted with any DNA-binding protein of interest.
3. Verify that the recombinant protein exhibits solubility and stability under low-salt binding conditions.

### DNA considerations

4. Use high-quality, protein-free genomic DNA with an A_260_/A_280_ ratio approximating 1.8.
5. Optimize DNase I digestion conditions to consistently generate 50–200 bp fragments, which are essential for high-resolution motif discovery.

### Controls (strongly recommended)

6. Input genomic DNA (no protein pull-down)
7. MBP-tagged protein without the TF of interest (e.g., MBP-only)
8. A related TF with known binding specificity (e.g., FOXP2 for FOXP3 studies)
9. Synthetic double-stranded oligonucleotides containing predicted binding sites to validate direct interactions prior to genome-wide PD-seq.

## Key resources table

**Table:** 

REAGENT or RESOURCE	SOURCE	IDENTIFIER
**Bacterial and virus strains**		

BL21 (DE3)	Stratagene	Cat#230130

**Chemicals, peptides, and recombinant proteins**		

Ampicillin sodium salt	Merck	Cat#BP785
Amylose resin	NEB	Cat#E8021L
Acryl/Bis 40% solution	Thermo Fisher Scientific	Cat#HC2040
APS	Thermo Fisher Scientific	Cat#HC2005
Agarose	Thermo Fisher Scientific	Cat#16500500
Acetic Acid Glacial	Thermo Fisher Scientific	Cat#039745.AE
Absolute ethyl alcohol	Merck	Cat#E7023
Boric acid	Thermo Fisher Scientific	Cat#A16624.0 B
DNase I	ZYMO RESEARCH	Cat#E1010
DTT	Merck	Cat#646563
EDTA·2H_2_O	Thermo Fisher Scientific	Cat#15576028
IPTG	Thermo Fisher Scientific	Cat#15529019
Imidazole	Merck	Cat#I202
LB premix	Thermo Fisher Scientific	Cat#12780052
LB Agar premix	Thermo Fisher Scientific	Cat#22700041
MgCl_2_·6H_2_O	Thermo Fisher Scientific	Cat#AM9530G
Ni-NTA agarose	QIAGEN	Cat#30210
Proteinase K	NEB	Cat#P8107S
PBS	Merck	Cat#P2272
Protease Inhibitor Cocktail Powder	Thermo Fisher Scientific	Cat#78429
PMSF	Merck	Cat#93482
SPRIselect	Beckman Coulter	Cat#B23317
SYBR Gold	Thermo Fisher Scientific	Cat#S11494
SYBR Green I	Thermo Fisher Scientific	Cat#S7567
TCEP	Merck	Cat#646547
Ultra Low Range DNA Ladder	NEB	Cat#N0558S
1Kb DNA Ladder	Thermo Fisher Scientific	Cat#10787026
100bp DNA Ladder	Thermo Fisher Scientific	Cat#15628019
100% Glycerol	Merck	Cat#G7893
1 M Tris-HCl	Thermo Fisher Scientific	Cat#15567027
5 M NaCl	Thermo Fisher Scientific	Cat#A57006

**Critical commercial assays**		

Qiagen Blood & Cell Culture DNA Maxi Kit	QIAGEN	Cat#13343
QIAquick PCR & Gel Cleanup Kit	QIAGEN	Cat#28506
NEBNext Ultra II DNA Library Prep with Sample Purification Beads	NEB	Cat#E7103S
NEBNext Multiplex Oligos for Illumina	NEB	Cat#E7600S

**Experimental models: Cell lines**		

EL4	ATCC	TIB-39

**Oligonucleotides**		

IR-FKHM-F: ATGGCGCTTGAACTTCTGTTTACTA CAGTAAACAGTCGGCTGGGAGAACA	IDTDNA	https://www.idtdna.com
IR-FKHM-R: TGTTCTCCCAGCCGACTGTTTACTGT AGTAAACAGAAGTTCAAGCGCCAT	IDTDNA	https://www.idtdna.com
FKHM-F: ATGGCGCTTGAACTTCTGTTTACCAA GGCGTGGAGTCGGCTGGGAGAACA	IDTDNA	https://www.idtdna.com
FKHM-R: TGTTCTCCCAGCCGACTCCACGCCTT GGTAAACAGAAGTTCAAGCGCCAT	IDTDNA	https://www.idtdna.com
No-FKHM-F: ATGGCGCTTGAACTTCCGAGAGG CAAGGCGTGGAGTCGGCTGGGAGAACA	IDTDNA	https://www.idtdna.com
No-FKHM-R: TGTTCTCCCAGCCGACTCCACGCC TTGCCTCTCGGAAGTTCAAGCGCCAT	IDTDNA	https://www.idtdna.com

**Recombinant DNA**		

His-MBP-FOXP3(N)	His-MBP-H3C-pMAL	N/A

**Other**		

HiTrap Heparin column	Cytiva	Cat#17040701
Amicon Ultra Centrifugal Filter	Millipore	Cat#UFC803024
Thermal cycler	BIO-RAD	Cat#1851196
Gel imager	Tanon	Cat#24TULRGB-12097
NanoDrop	Thermo Fisher Scientific	Cat#840-317500
Water bath	YIHENG	DK-8D
Shaking incubator	Scilogex	SLK-O3000-S
Centrifuge	Beckman Coulter	JXN-26
Centrifuge rotor	Beckman Coulter	JLA-8.1
Centrifuge tube	Beckman Coulter	Cat#C31597
0.22 μm filter	Merck	Cat#SLGPR33RB

## Materials and equipment

**Table:** 5 M Imidazole

Reagent	Final concentration	Amount
Imidazole	5 M	17.02 g
ddH_2_O	N/A	to 50 mL
Total	N/A	50 mL

Store at 4°C for up to six months.

**Table:** 0.5 M TCEP

Reagent	Final concentration	Amount
TCEP	0.5 M	1.433 g
ddH_2_O	N/A	to 10 mL
Total	N/A	10 mL

Rotate the solution at 25°C for at least 1 h to ensure complete dissolution. Sterilize using 10 mL syringe with syringe filter into 15 mL conical tube. Aliquot 1 mL and store at −20°C for up to a year.

**CRITICAL:** Always wear gloves and operate in a fume hood.

**Table:** 100 mg/mL ampicillin sodium salt

Reagent	Final concentration	Amount
Ampicillin sodium salt	100 mg/mL	1 g
ddH_2_O	N/A	to 10 mL
Total	N/A	10 mL

Sterilize using 50 mL syringe with syringe filter into 50 mL conical tube. Aliquot 1 mL and store at −20°C for up to six months.

**Table:** LB

Reagent	Final concentration	Amount
LB premix	25 g/L	25 g
ddH_2_O	N/A	to 1 L
Total	N/A	1 L

Mix the above components in an autoclavable flask and autoclave. Store at 4°C for up to two months.

**Table:** LB agar supplemented with ampicillin

Reagent	Final concentration	Amount
LB agar premix	40 g/L	40 g
Ampicillin sodium salt (100 mg/mL)	100 μg/mL	1 mL
Chloramphenicol (34 mg/mL)	34 μg/mL	1 mL
ddH_2_O	N/A	to 1L
Total	N/A	1L

Mix all components except ampicillin sodium salt and chloramphenicol in an autoclavable flask and autoclave. Allow to cool to 60°C, then add the ampicillin sodium salt and chloramphenicol solution. Pour into Petri dishes and store at 4°C for up to two months.

**Table:** Annealing buffer

Reagent	Final concentration	Amount
Tris-HCl pH 7.5 (1 M)	10 mM	500 μL
NaCl (5 M)	50 mM	500 μL
ddH_2_O	N/A	to 50 mL

Store at 25°C for up to 1 year.

**Table:** Protein lysis buffer

Reagent	Final concentration	Amount
Tris-HCl pH 7.5 (1 M)	50 mM	2.5 mL
NaCl (5 M)	300 mM	3 mL
100% Glycerol	5%	2.5 mL
Imidazole (5 M)	10 mM	100 μL
TCEP (0.5 M)	2 mM	200 μL
PMSF (100 mM)	1 mM	500 μL
ddH_2_O	N/A	to 50 mL
Total	N/A	50 mL

Store at 4°C for up to six months. Add TCEP and PMSF freshly.

**Table:** Protein wash buffer

Reagent	Final concentration	Amount
Tris-HCl pH 7.5 (1 M)	50 mM	2.5 mL
NaCl (5 M)	1 M	10 mL
100% Glycerol	5%	2.5 mL
Imidazole (5 M)	50 mM	500 μL
TCEP (0.5 M)	2 mM	200 μL
ddH_2_O	N/A	to 50 mL
Total	N/A	50 mL

Store at 4°C for up to six months. Add TCEP freshly.

**Table:** Protein elution buffer

Reagent	Final concentration	Amount
Tris-HCl pH 7.5 (1 M)	50 mM	2.5 mL
NaCl (5 M)	1 M	10 mL
100% Glycerol	5%	2.5 mL
Imidazole (5 M)	200 mM	2 mL
TCEP (0.5 M)	2 mM	200 μL
ddH_2_O	N/A	to 50 mL
Total	N/A	50 mL

Store at 4°C for up to six months. Add TCEP freshly.

**Table:** Heparin buffer A

Reagent	Final concentration	Amount
Tris-HCl pH 7.5 (1 M)	20 mM	4 mL
NaCl (5 M)	50 mM	2 mL
DTT (1 M)	2 mM	400 μL
ddH_2_O	N/A	to 200 mL
Total	N/A	200 mL

Store at 4°C for up to six months. Add DTT freshly.

**Table:** Heparin buffer B

Reagent	Final concentration	Amount
Tris-HCl pH 7.5 (1 M)	20 mM	4 mL
NaCl (5 M)	1 M	40 mL
DTT (1 M)	2 mM	400 μL
ddH_2_O	N/A	to 200 mL
Total	N/A	200 mL

Store at 4°C for up to six months. Add DTT freshly.

**Table:** Incubation buffer

Reagent	Final concentration	Amount
Tris-HCl pH 7.5 (1 M)	20 mM	1 mL
NaCl (5 M)	100 mM	1 mL
MgCl_2_·6H_2_O (1 M)	1.5 mM	75 μL
ddH_2_O	N/A	to 50 mL
Total	N/A	50 mL

Store at 4°C for up to six months.

**Table:** 10×TBE buffer

Reagent	Final concentration	Amount
Tris base	108 g/L	108 g
Boric acid	55 g/L	55 g
EDTA·2H_2_O	7.44 g/L	7.44 g
ddH_2_O	N/A	to 1 L
Total	N/A	1 L

Rotate the solution at 25°C for 12 h to ensure complete dissolution. Store at 25°C for up to six months.

**Table:** 1×TBE buffer

Reagent	Final concentration	Amount
10×TBE buffer	1×	100 mL
ddH_2_O	N/A	to 1 L
Total	N/A	1 L

Prepare the buffer before use.

**Table:** 10% TBE gel

Reagent	Final concentration	Amount
AB (40%)	10%	2.5 mL
APS (10%)	0.1%	100 μL
1×TBE	N/A	to 10 mL
Total	N/A	10 mL

Prepare it before use.

**CRITICAL:** Always wear gloves and operate in a fume hood.

**Table:** Digestion buffer

Reagent	Final concentration	Amount
Tris-HCl pH 7.5 (1 M)	20 mM	1 mL
NaCl (5 M)	50 mM	500 μL
MgCl_2_·6H_2_O (1 M)	1.5 mM	75 μL
ddH_2_O	N/A	to 50 mL
Total	N/A	50 mL

Store at 4°C for up to six months.

**Table:** 50×TAE buffer

Reagent	Final concentration	Amount
Tris base	242 g/L	242 g
Glacial acetic acid	N/A	57.1 mL
EDTA·2H_2_O	37.2 g/L	37.2 g
ddH_2_O	N/A	to 1 L
Total	N/A	1 L

Rotate the solution at 25°C for 12 h to ensure complete dissolution. Store at 25°C for up to six months.

**Table:** 1×TAE buffer

Reagent	Final concentration	Amount
50×TAE buffer	1×	20 mL
ddH_2_O	N/A	to 1 L
Total	N/A	1 L

Prepare the buffer before use.

**Table:** 2% Agarose gel

Reagent	Final concentration	Amount
Agarose	2%	1 g
1×TAE buffer	N/A	50 mL
Nucleic acid dye	1×	5 μL
Total	N/A	50 mL

Prepare it before use.

**CRITICAL:** Always wear gloves and operate in a fume hood.

***Alternatives:*** This protocol uses QIA quick PCR & Gel Cleanup Kit (Qiagen #28506) to purify the DNA. If QIAquick PCR & Gel Cleanup Kit is unavailable, similar clean-up kits from other suppliers (e.g., Thermo Fisher PureLink Quick Gel Extraction and PCR Purification Kit, or equivalent products from Promega, Zymo Research, etc.) can be used with comparable buffer volumes and centrifugation conditions, following the manufacturer's instructions.

***Alternatives:*** This protocol uses Qiagen Blood & Cell Culture DNA Maxi Kit (Qiagen #13343) to extract genomic DNA. If Qiagen Blood & Cell Culture DNA Maxi Kit is unavailable, similar genomic DNA extraction kits from other suppliers (e.g., Promega Wizard Genomic DNA Purification Kit, Thermo Fisher GeneJET Genomic DNA Purification Kit, or equivalent products from other vendors) can be used with comparable buffer volumes and centrifugation conditions, following the manufacturer's instructions.

***Alternatives:*** This protocol uses DNase I to digest genomic DNA. Mechanical shearing (e.g., using Covaris) provides more uniform fragments and minimizes the risk of over-digestion. The method can be selected based on specific laboratory conditions. If using Covaris, parameters should be optimized to obtain fragments ranging from 50 to 200 bp.

***Alternatives:*** This protocol uses NEBNext Ultra II DNA Library Prep Kit with Sample Purification Beads (NEB #E7103S) to construct library. If NEBNext Ultra II DNA Library Prep Kit is unavailable, optimized library building kits are available for all major sequencing platforms or service providers.

***Alternatives:*** This protocol uses a Bioanalyzer to assess the extent of DNA fragmentation.

Alternatives are for example the Agilent TapeStation. The TapeStation offers more flexibility in the number of samples that can be analyzed at once.

## Step-by-step method details

### Recombinant protein expression

**Timing: 2 days**

This section details the expression of the recombinant TF, bearing various affinity tags, using an Escherichia coli system. As exemplified by FOXP3, the general method also refers to Zhang et al.^1^

1. Transform the competent BL21 (DE3) cells with the pMAL plasmid carrying the His-MBP-FOXP3(ΔN) coding sequences.
  a. Thaw competent cells on ice.
  b. Add 5 μL of plasmid into the competent cells (50 μL).
  c. Gently tap the bottom of the tube about 2-3 times, and immediately incubate it on ice for 15 min.
  d. Heat-shock the competent cells at 42°C for 45 s in a water bath.
  e. Place the competent cells on ice for 3 min.
  f. Add 1 mL of sterile 37°C pre-warmed LB medium to the tube, invert several times, and resuscitate at 37°C shaker for 1 h with shaking (220 rpm).
  g. Spread the culture onto an ampicillin and chloramphenicol LB agar plate and incubate at 37°C for 12 h.
2. Pick a single colony from the LB agar plate. Inoculate the colony into 10 mL of sterile LB medium supplemented with 100 μg/mL of ampicillin and 34 μg/mL chloramphenicol.
3. Culture the cells on large scale and induce protein expression.
  a. Dilute the culture 1:100 into 1 L of sterile LB medium with antibiotic.
  b. Culture the cells in a shaking incubator at 220 rpm and 37°C until the optical density at 600 nm (OD600) exceeds 0.6–0.8.
  c. Cool in the refrigerator for 20 min.
  d. Add IPTG at a final concentration of 0.2 mM to induce protein expression.
  e. Incubate at 18°C for 16–20 h with shaking (220 rpm).
4. Harvest the cells.
  a. Centrifuge at 4,000 × g for 20 min at 4°C and discard the supernatant.
  b. Resuspend the cell pellet in 40 mL of PBS.
  c. Transfer to a 50 mL centrifuge tube.
  d. Centrifuge at 4,000 g for 20 min at 4°C and discard the supernatant. Cell pellets can be stored at −80°C.

### Protein purification

**Timing: 2 days**

**Figure 1:**
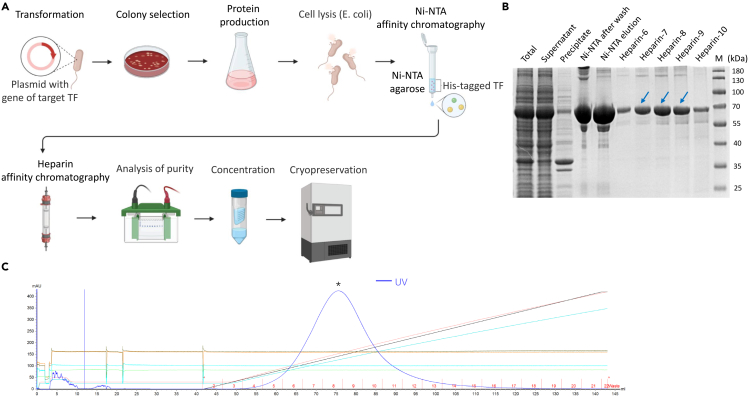
Expression and purification of recombinant His-MBP-FOXP3(ΔN) protein (A) Schematic flowchart of the protein purification process, including Ni-NTA affinity chromatography followed by HiTrap Heparin HP chromatography. (B) SDS-PAGE analysis of purified fractions during the HiTrap Heparin HP chromatography. The arrow indicates the target protein. M represents the molecular weight marker, with numbers on the right indicating molecular weights in kDa. The gel was stained with Coomassie Blue dye. (C) The HiTrap Heparin HP affinity chromatography profile of the final purified protein. An asterisk indicates the peak corresponding to the desired FOXP3.

The following steps detail the purification of recombinant TFs (Figure 1A). We advise first-time users to characterize samples from each stage, including SDS-PAGE analysis with Coomassie brilliant blue staining and western blotting, to confirm expression, molecular weight, and purity.

5. Thaw the pellet and resuspend it in 40 mL of protein lysis buffer by rotation at 4°C.
6. Lyse the cells.
  a. Lyse cells by high-pressure homogenization using the Emulsiflex C3 system at 4°C.
  b. Centrifuge the lysate at 10,000 × *g* for 30 min at 4°C to remove cellular debris.
7. Perform affinity chromatography using Ni-NTA agarose.
  a. Load the clarified supernatant onto a chromatography column with pre-equilibrated Ni-NTA agarose with protein lysis buffer and incubate for 1 to 2 h. Turn on the column switch and the liquid flows out naturally.
  b. Wash the non-specific proteins with 5 column volumes (CVs) of low-concentration imidazole protein wash buffer three times.
  c. Elute the His-tagged protein of interest with 1CV high concentration of imidazole protein elution buffer five times.

***Note:*** The His tag fused to the protein of interest specifically binds to Ni-NTA. In this step, proteins that do not have the His tag are removed. Imidazole in protein elution buffer binds more strongly to Ni-NTA, resulting in the elution of the His-tagged protein of interest.

8. Perform affinity chromatography using the HiTrap Heparin column.
  a. Load the protein onto a HiTrap Heparin column.
  b. Wash away impurities with the low salt concentration heparin buffer A.
  c. Elute the protein of interest with the buffer B that gradually increases from low to high salt concentration (Figure 1B).

***Note:*** Heparin chromatography effectively purifies DNA-binding proteins, coagulation factors, and other plasma proteins with high resolution. The recombinant TF binds to the heparin matrix, and impurities are efficiently removed by washing with low-salt buffer, significantly enhancing target protein purity. The eluted protein peak is collected and analyzed by SDS-PAGE followed by Coomassie brilliant blue staining and western blotting to confirm expression, molecular weight, and purification efficiency (Figure 1B).

9. Concentrate the protein, aliquot, flash-freeze in liquid N_2_, and store at −80°C.

***Note:*** Check protein purity and concentration by SDS-PAGE and a Bradford assay before proceeding.

### Preliminary test of binding affinity using synthetic DNA

**Timing: 2–3 h**

To directly probe the interaction between TFs and DNA in a controlled in vitro setting, we developed an approach that combines protein-DNA binding assays with next-generation sequencing (NGS). This strategy enables genome-wide identification of intrinsic DNA-binding specificity. As a preliminary validation, we first employ synthetic DNA sequences containing defined motifs^9^ to assess binding specificity in vitro before proceeding to genome-wide PD-seq.

10. Prepare DNA oligos.
  a. Design and synthesize DNA oligos with specific sequences that have been demonstrated with diverse binding affinity with FOXP3.
  b. Mix forward and backward single-strand DNA in equal proportions.
  c. Anneal for double-stranded DNA using a thermal cycler following the steps below.

**Table:** 

Temperature	Time
95°C	5 min
90°C	1 min
90°C → 25°C	−5°C/1 min
4°C	Hold

***Note:*** For FOXP3, use IR-FKHM, an inverted-repeat forkhead motif (FKHM), as the positive control because it exhibits very strong and specific binding to FOXP3. Use FKHM (TGTTTCA) as a moderate binder representing the canonical consensus motif, and no-FKHM as the negative control, as it has been experimentally validated to show no detectable FOXP3 binding. Oligos should be ordered in dry form and dissolved in annealing buffer to a final concentration of 100 μM. Successful annealing of dsDNA can be confirmed by TBE gel electrophoresis, which should show a single band of the expected size.

11. Incubate the protein and DNA.
  a. Mix His-MBP-FOXP3 protein (final concentration: 0.4 μM) with each dsDNA oligo of the indicated sequence (final concentration: 0.1 μM) in incubation buffer to a total volume of 200 μL.
  b. Incubate at 25°C for 20 min with gentle rotation.
12. FOXP3 pull down.
  a. Add equilibrated amylose resin to the mixture.
  b. Incubate at 4°C for 1 h with rotation to capture the protein-DNA complexes.
  c. Pellet the resin by gentle centrifugation at 2,000 × g for 1 min.
  d. Remove the supernatant carefully.

***Note:*** The amylose resin should be equilibrated with incubation buffer.

13. Wash amylose resin mixture 3 times with 1 mL incubation buffer by centrifugation at 4,000 × g for 1 min to remove non-specifically bound DNA.

**CRITICAL:** Perform washes thoroughly but gently to avoid losing resin or disrupting specific interactions.

14. Release bound DNA by adding Proteinase K (0.5 mg/mL) to digest the protein and incubating at 55°C for 30-60 min.
15. Analyze the binding affinity of the protein and DNA.
  a. Recover the supernatant containing DNA that are bound by FOXP3 by using QIAquick PCR & Gel Cleanup Kit (Qiagen #28506) according to manufacturer’s instructions.
  b. Load the mixture into the wells of a 10% TBE gel in an electrophoresis tank filled with 1×TBE buffer.
  c. Run the gel at 150 V and 4°C until the dye line is approximately 90% of the gel.
  d. Stain the gel 5 min with SYBR Gold (1:10000 dilution with ddH2O) at 25°C on the shaker.
  e. Visualize SYBR Gold-stained DNA bands using a gel imaging system.

**Figure 2:**
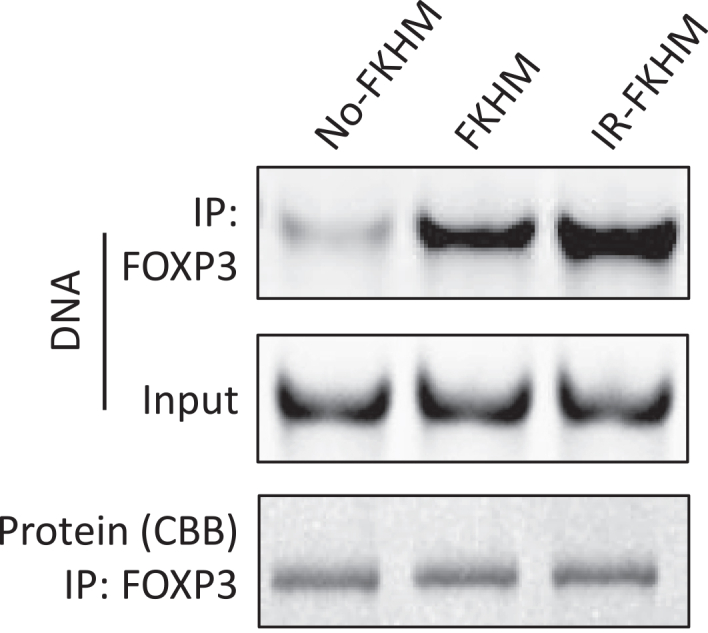
Validation of FOXP3 binding specificity using synthetic oligonucleotides SYBR Gold-stained gel image of DNA recovered from pull-down assays with different oligos. FOXP3 specifically binds to the oligos containing the FKHM and IR-FKHM, while showing much weaker binding to oligo with no-FKHM. CBB represents the Coomassie brilliant blue staining.

***Note:*** If QIAquick PCR & Gel Cleanup Kit is unavailable, similar DNA clean-up kits from Thermo Fisher (PureLink Quick Gel Extraction and PCR Purification Kit) can be used with comparable buffer volumes and centrifugation conditions. Stronger DNA bands indicate higher binding affinity (e.g., IR-FKHM and FKHM), whereas weak or undetectable bands reflect low affinity (e.g., no-FKHM) (Figure 2).

Based on successful recapitulation of FOXP3-DNA binding specificity in vitro using synthetic oligonucleotides, we next utilize fragmented and naked genomic DNA for discovering its intrinsic binding sites genome-wide.

### Genomic DNA extraction

**Timing: 4–6 h**

**Figure 3:**
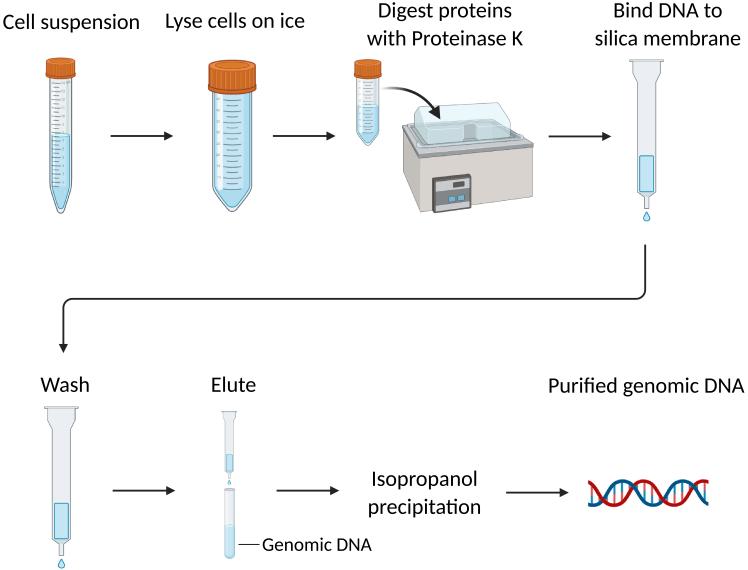
Genomic DNA extraction Schematic flowchart of the Genomic DNA Extraction process. Schematic flowchart illustrating the major steps of genomic DNA extraction from cells using the Qiagen Blood & Cell Culture DNA Maxi Kit. The workflow includes cell lysis, protein digestion with Proteinase K, binding of genomic DNA to the column, washing steps, and elution of purified DNA. The final step yields high-quality, protein-free genomic DNA with an A260/A280 ratio of approximately 1.8, suitable for downstream fragmentation and PD-seq analysis.

***Note:*** If Qiagen Blood & Cell Culture DNA Maxi Kit is unavailable, similar genomic DNA extraction kit from Promega (Wizard Genomic DNA Purification Kit) or Thermo Fisher (GeneJET Genomic DNA Purification Kit) can be used with comparable buffer volumes and centrifugation conditions.

16. Isolate naked genomic DNA from EL4 cells using Qiagen Blood & Cell Culture DNA Maxi Kit (Qiagen #13343) according to manufacturer’s instructions (Figure 3).

**CRITICAL:** Test DNA integrity by agarose gel electrophoresis; Ensure DNA is largely free of RNA and protein contamination by spectrophotometry (A_260_/A_280_ ratio ∼1.8); Obtain as much high-quality genomic DNA as possible to ensure sufficient input for the subsequent pull-down assay.

### Genomic DNA fragmentation

**Timing: 1 h**

Optimal digestion conditions and duration are dependent on the cell line and must be optimized empirically.

17. DNase I Fragmentation.
  a. Mix 50 μg of genomic DNA with 8 μL of DNase I in digestion buffer to a total volume of 200 μL.
  b. Incubate at 25°C for 3 to 4 min. troubleshooting 1.
  c. Add 0.5% SDS and 5 mM EDTA to stop the reaction.

**CRITICAL:** It is strongly advised to initially optimize digestion conditions using a small-scale pilot reaction (e.g., 5 μg of genomic DNA). Empirically titrate incubation time to yield fragments predominantly between 50–200 bp, as assessed by agarose gel electrophoresis. Once optimal conditions are determined, scale up the reaction (e.g., 50 μg). Over-digestion produces excessively short fragments, complicating library construction; under-digestion yields overly long fragments, reducing specificity.

***Note:*** Mechanical shearing (e.g., using Covaris) provides more uniform fragments and minimizes the risk of over-digestion. The method can be selected based on specific laboratory conditions. If using Covaris, parameters should be optimized to obtain fragments ranging from 50 to 200 bp.

**Figure 4:**
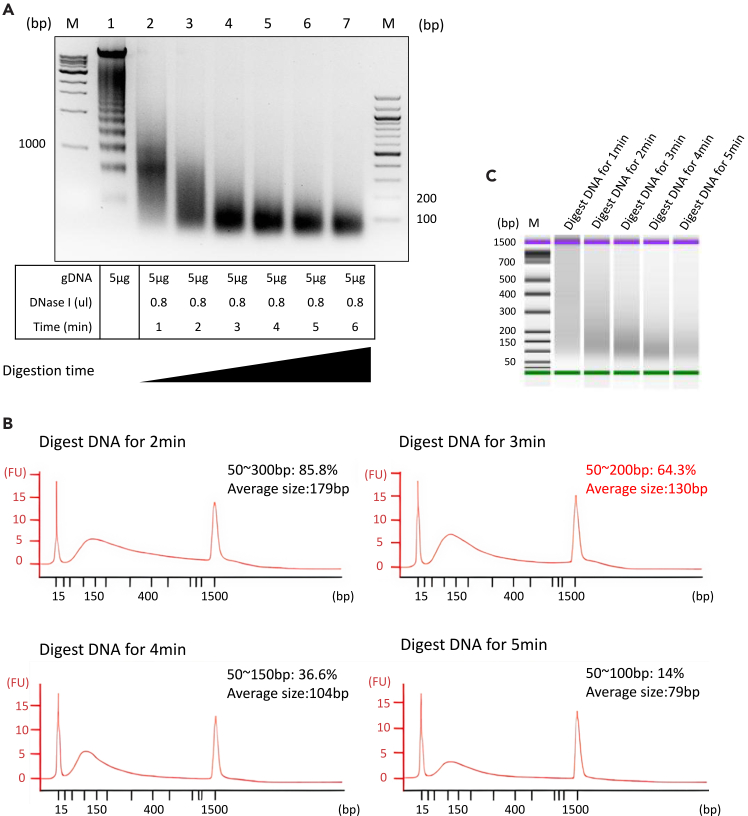
Fragmentation of genomic DNA (A) Agarose gel electrophoresis of genomic DNA before (lane 1) and after (lane 2-7) DNase I digestion with different incubation time. Successful digestion results in a smear of fragments ranging from 50 to 200 bp. M represents the DNA ladder, with numbers on the side indicating base pair in bp. (B) Electropherogram from the Agilent Bioanalyzer showing the size distribution of the DNase I-treated genomic DNA. After digestion for 3 min, 64.3% of the fragments are 50∼200 bp, and average size is 130 bp. (C) The virtual gel image corresponding to the electropherogram in (B). M represents the DNA ladder, with numbers on the left indicating base pair in bp.
  b. Excise the gel slice corresponding to 50–150 bp using a clean scalpel. Use a DNA ladder as a size guide.
  c. Purify DNA from the gel slice using a gel extraction kit (e.g., QIAquick PCR & Gel Cleanup Kit, Qiagen #28506) according to the manufacturer’s instructions.

***Note:*** If gel extraction is not feasible or higher throughput is desired, enrich 50–200 bp fragments using SPRIselect beads (Beckman Coulter #B23317) with double-sided size selection. The bead-to-sample ratio determines the fragment size range that binds to the beads. Higher ratios capture smaller fragments, while lower ratios capture larger fragments. For 50–200 bp fragments, use 0.9× beads (relative to sample volume) in the first round to remove fragments >200 bp, then add additional beads to the supernatant to reach 1.2× (relative to original sample volume) to capture fragments ≥50 bp. Elute in 30–50 μL of 0.1× TE or 10 mM Tris-HCl (pH 8.0). These ratios are starting points based on the SPRIselect User Guide and should be validated experimentally for your specific fragment size distribution. If your target fragment size range differs, refer to the SPRIselect User Guide to determine the appropriate bead ratios for your specific application. If SPRIselect is unavailable, AMPure XP beads (Beckman Coulter #A63881) can be used with similar ratios; refer to the manufacturer's instructions for optimization.

18. Purification of fragmented DNA.
  a. Load the digested DNA onto a 2% agarose gel and run electrophoresis until fragments are well separated (Figure 4A).

**Pause point**: Purified DNA fragments can be stored at −20°C for several weeks.

### Bioanalyzer analysis

**Timing: 1 h**

19. The Agilent Bioanalyzer High Sensitivity DNA chip (or an equivalent system such as the Agilent TapeStation) is employed for quality control,^10^^,^^11^ validating the size distribution of gDNA fragments against agarose gel analysis to confirm their suitability for protein pull-down assays (Figures 4B and 4C).

### FOXP3 pull-down using fragmented genomic DNA

**Timing: 2–3 h**

The recovered fragmented genomic DNA proceeds directly to the pull-down assay. The use of genomic DNA, as opposed to synthetic DNA oligos, enables the testing of sequence specificity in the context of a naturally existing repertoire of sequences.

20. Incubate the protein and DNA.
  a. Mix 0.4 μM FOXP3 with 0.1 μM fragmented genomic DNA in incubation buffer to a total volume of 200 μL.
  b. Incubate for 20 min at 25°C with gentle rotation.
  c. TIP: Include control reactions: (1) input (2) MBP-only protein + gDNA.
21. Perform pull-down as step 12–14 in preliminary test of binding affinity with synthetic DNA (Figure 5). troubleshooting 2.

**Figure 5:**
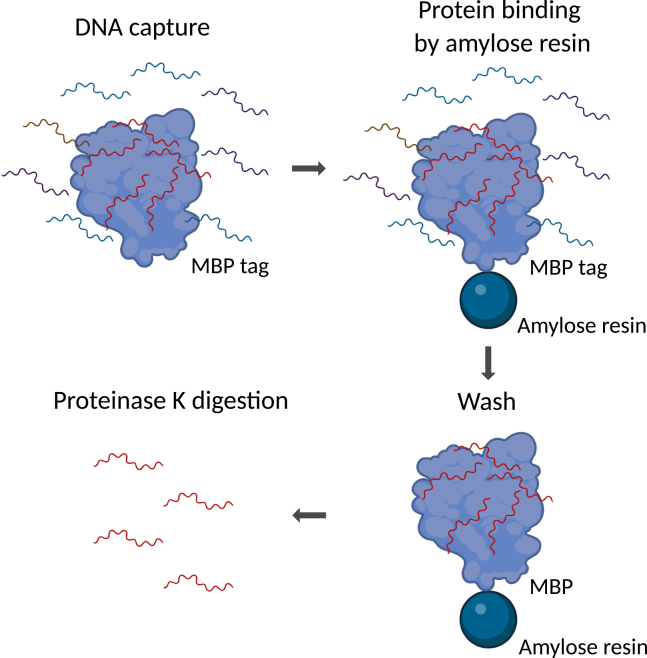
Workflow of the genomic DNA pull-down assay Schematic diagram illustrating the major steps of the pull-down assay using fragmented genomic DNA and MBP-tagged recombinant protein. (1) DNA capture: Fragmented genomic DNA (50-200 bp) is incubated with the MBP-tagged recombinant protein, allowing DNA binding. (2) Protein binding by amylose resin: The MBP-tagged transcription factor (e.g., FOXP3) is captured by amylose resin via the MBP tag. (3) Wash: Unbound and non-specifically bound DNA fragments are removed through multiple washes. (4) Proteinase K digestion: The protein-DNA complexes are digested with Proteinase K to release the bound DNA fragments.
22. Use the QIAquick PCR & Gel Cleanup Kit to purify DNA.
23. Quantify the recovered DNA by using Qubit fluorometer. troubleshooting 3.

### Library preparation and sequencing

**Timing: 1 day**

**Figure 6:**
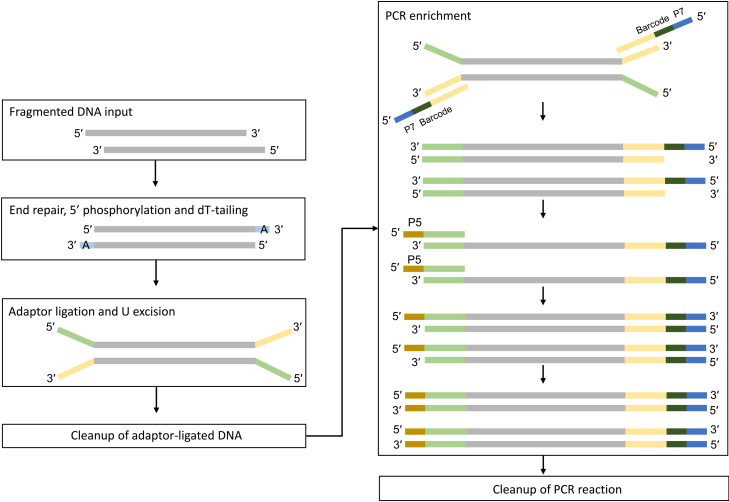
Library preparation Schematic diagram illustrating the major steps of library construction using the NEBNext Ultra II DNA Library Prep Kit. The workflow includes. (1) end repair and dA-tailing of fragmented genomic DNA. (2) adaptor ligation (hairpin adaptor containing dU); USER enzyme cleavage to open the adaptor, and (3) PCR enrichment with indexed primers to generate the final sequencing library.

Construct sequencing libraries from the recovered PD-seq DNA using the NEBNext Ultra II DNA Library Prep Kit for Illumina with Sample Purification Beads (NEB #E7103S) and NEBNext Multiplex Oligos for Illumina (NEB #E7600S) following the manufacturer’s instructions with the settings below. The key steps are outlined in Figure 6.

***Note:*** The NEBNext Ultra II DNA Library Prep Kit is available in two versions. NEB #E7103S includes purification beads and was used in this protocol. If using the bead-free version (NEB #E7645S), SPRIselect (Beckman Coulter #B23317) or AMPure XP beads (Beckman Coulter #A63881) must be purchased separately and used for all bead-based cleanup steps.

24. NGS library preparation.
  a. End preparation.
    i. Dilute the recovered PD-seq DNA to 30 ng in 50 μL using 0.1× TE (10 mM Tris-HCl, pH 8.0, 0.1 mM EDTA) or 10 mM Tris-HCl (pH 8.0). If the concentration is too low, use the maximum available volume and adjust to 50 μL with the same buffer.
    ii. In a sterile nuclease-free PCR tube on ice, combine the following components:

**Table:** 

Component	Volume
Pull-down DNA (30 ng)	50 μL
NEBNext Ultra II End Prep Reaction Buffer	7 μL
NEBNext Ultra II End Prep Enzyme Mix	3 μL
Total	60 μL
    iii. Mix thoroughly by pipetting up and down at least 10 times (set pipette to 50 μL). Perform a quick spin to collect all liquid from the sides of the tube.
    iv. Place in a thermal cycler with the heated lid set to ≥75°C and run the following program:

**Table:** 

Temperature	Time
20°C	30 min
65°C	30 min
4°C	Hold
      ***Note:*** Pull-down typically yields sufficient DNA for library construction. Calibrate the starting DNA amount for all samples (Input gDNA fragments, MBP pull-down DNA, and FoxP3 pull-down DNA) to 30 ng per library. Process all samples in duplicate.
  b. Adaptor ligation.
    i. Dilute the NEBNext Adaptor for Illumina (found in NEBNext Multiplex Oligos for Illumina) 10-fold (1:10) in 10 mM Tris-HCl (pH 7.5-8.0) with 10 mM NaCl. Prepare fresh before use.

**Table:** 

Input	Adaptor dilution	Working adaptor concentration
101 ng-1 μg	No dilution	15 μM
5 ng-100 ng	10-fold (1:10)	1.5 μM
Less than 5 ng	25-fold (1:25)	0.6 μM
    ii. Add the following components directly to the End Prep Reaction Mixture (60 μL from Step End preparation):

**Table:** 

Component	Volume
End Prep Reaction Mixture	60 μL
NEBNext Adaptor for Illumina (1:10 diluted)	2.5 μL
NEBNext Ultra II Ligation Master Mix	30 μL
NEBNext Ligation Enhancer	1 μL
Total	93.5 μL
    iii. Mix thoroughly by pipetting up and down at least 10 times (set pipette to 80 μL). Perform a quick spin to collect all liquid.
    iv. Incubate at 20°C for 15 min in a thermal cycler with the heated lid off.
    v. Add 3 μL of USER Enzyme to the ligation mixture. Mix well and incubate at 37°C for 15 min with the heated lid set to ≥47°C.
      **CRITICAL:** Select the appropriate adaptor dilution based on input DNA amount to minimize adapter-dimer formation. The dilutions provided here are a general starting point. The appropriate adaptor dilution for your sample input and type may need to be optimized experimentally. Incorporating a novel hairpin loop structure, the NEBNext Adaptor ligates with increased efficiency to end-repaired, dA-tailed DNA. The loop contains a U, which is removed by treatment with USER™ Enzyme (a mix of UDG and Endo VIII), to open up the loop and make it available as a substrate for PCR. During PCR, barcodes can be incorporated by use of the NEBNext index primers, thereby enabling multiplexing.
      **Pause point**: Samples can be stored 12–16 h at −20°C.
  c. Cleanup of adaptor-ligated DNA.
    i. Vortex NEBNext Sample Purification Beads to resuspend completely. Allow beads to warm to 25°C for at least 10 min before use.
    ii. Add 87 μL (0.9×) of resuspended beads to the adaptor-ligation reaction (93.5 μL). Mix well by pipetting up and down at least 10 times. Ensure the mixture is homogeneous.
    iii. Incubate at 25°C for 5 min.
    iv. Place the tube on a magnetic stand. Wait until the solution is clear (approximately 5 min), then carefully remove and discard the supernatant. Do not disturb the beads.
    v. While on the magnet, add 200 μL of freshly prepared 80% ethanol. Incubate for 30 s, then remove and discard the ethanol.
    vi. Repeat the ethanol wash once for a total of two washes. After the second wash, remove all visible ethanol. If necessary, briefly spin the tube, place back on the magnet, and remove residual ethanol with a P10 pipette tip.
    vii. Air-dry the beads for up to 5 min with the lid open. Do not over-dry—the beads should remain dark brown and glossy, not cracked or lighter brown.
    viii. Remove the tube from the magnetic stand. Elute the DNA by adding 17 μL of 10 mM Tris-HCl or 0.1× TE. Mix thoroughly by pipetting up and down 10 times.
    ix. Incubate at 25°C for 2 min.
    x. Place the tube back on the magnetic stand. After the solution is clear (approximately 5 min), transfer 15 μL of eluate to a new PCR tube.
      **CRITICAL:** For input DNA ≤50 ng, do not perform size selection to maintain library complexity. Follow the cleanup protocol without size selection. If the starting material is > 50 ng, follow the manufacturer's instructions for size selection.
      **Pause point**: Samples can be stored at −20°C.
  d. PCR enrichment.
    i. Set up the PCR reaction in a sterile PCR tube on ice:

**Table:** 

Component	Volume
Adaptor-ligated DNA (from Step Cleanup of Adaptor-ligated DNA)	15 μL
NEBNext Ultra II Q5 Master Mix	25 μL
Index Primer/i7 Primer	5 μL
Universal PCR Primer/i5 Primer	5 μL
Total	50 μL
    ii. Mix thoroughly by pipetting up and down at least 10 times (set pipette to 40 μL). Perform a quick spin.
    iii. Place in a thermal cycler and run the following program:

**Table:** 

Cycle step	Temperature	Time	Cycles
Initial denaturation	98°C	30 s	1
Denaturation	98°C	10 s	4–5
Annealing/Extension	65°C	75 s	
Final Extension	65°C	5 min	1
Hold	4°C	–	–
      ***Note:*** Use only one i7 primer/ index primer per sample. Use only one i5 primer (or the universal primer for single index kits) per sample The choice of indexing strategy depends on the number of samples to be multiplexed and the sensitivity requirements of the experiment. Refer to the respective NEBNext Multiplex Oligos manual for valid barcode combinations and for guidance on pooling libraries for sequencing.
      **CRITICAL:** The setting of PCR cycle number needs to be adjusted according to the initial input amount and sample type. The ideal cycle number should meet two requirements: first, it should be sufficient to generate enough library fragments to ensure successful sequencing; second, it is necessary to avoid PCR artifacts or over-amplification caused by an excessively high cycle number. Usually, the recommended cycle number in the NEB operation manual can be used as the initial condition, and then optimized for standard library preparation samples on this basis.
  e. Cleanup of PCR reaction.
    i. Purify the PCR product following the same bead-based cleanup procedure described in Step Cleanup of Adaptor-ligated DNA, with the modifications: Use 45 μL (0.9×) of resuspended beads; Elute final library in 33 μL of 0.1× TE and transfer 30 μL to a new tube.
    ii. Proceed to Library Quality Control.
25. Library quality control.
  a. Quantification: Measure library concentration using Qubit fluorometer.
  b. Size distribution analysis: Assess library fragment size using an Agilent Bioanalyzer High Sensitivity DNA chip or TapeStation. Dilute samples before loading if necessary (Figures 7A and 7B).

**Figure 7:**
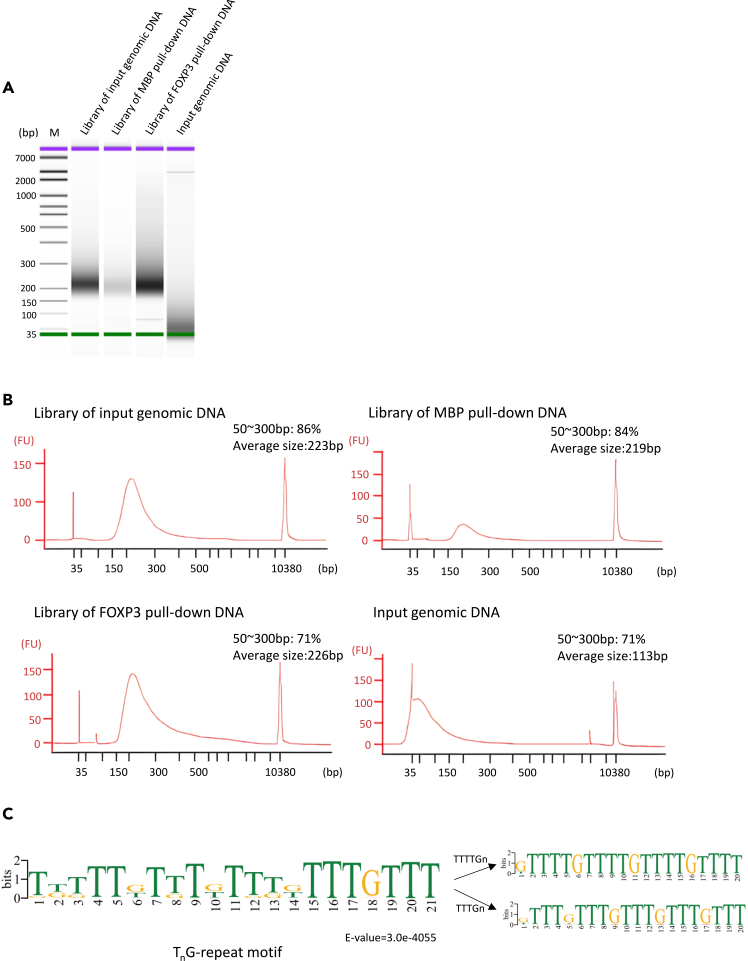
Library quality control and identification of FOXP3 intrinsic binding motif (A) Representative Bioanalyzer trace of the final sequencing library. The major peak corresponds to the adapter-ligated fragments ready for sequencing. (B) The virtual gel image corresponding to the electropherogram in (A). The DNA distribution of the libraries is mostly 170∼290 bp, implicating the successful adding of the 120 bp adaptor from input (50∼150 bp). (C) De novo motif identified from the FOXP3 PD-seq peaks using MEME. The most significantly enriched motif is a T_n_G microsatellite repeat, which is distinct from the canonical FKHM. This motif can be summarized as a combination of several simple tandem repeats, such as TTTG and TTTTG.

**CRITICAL:** After cleanup, validate library quality before sequencing. Expected results: final library size distribution of 170–290 bp (50–150 bp insert plus ∼120 bp adaptor sequences), average size approximately 220–230 bp, adapter-dimer contamination (<150 bp) <5% of total library, and >80% of fragments within the 50–300 bp range. If adapter-dimer peaks appear (>5% at ∼120 bp), consider increasing adaptor dilution (e.g., from 1:10 to 1:25) or performing an additional post-PCR SPRI cleanup in future experiments.

26. Sequencing.
  a. Pool libraries at equimolar concentrations based on Qubit quantification and Bioanalyzer analysis.
  b. Sequence on an Illumina platform (e.g., using a 75-cycle kit for 2×38 bp or a 100-cycle kit for 2×50 bp paired-end reads, aiming for 20-50 million reads per sample). troubleshooting 4.

**CRITICAL:** Ensure the chosen read length is shorter than the final library insert size to avoid sequencing through adapters. Pre-made library QC performed by the service provider should be consistent with Bioanalyzer results.

### Data analysis

**Timing: 2–3 days**

The bioinformatic analysis of PD-seq data follows a pipeline analogous to that used for ChIP-seq data.^11^ The following representative commands outline the key steps used in our analysis. File names, paths, genome builds, and parameters should be adjusted according to your specific experimental setup and computational environment.

The raw PD-seq sequencing data generated in this protocol have been deposited in the Gene Expression Omnibus (GEO) under accession number GEO: GSE243606 and are publicly available. Researchers can use this dataset to test and validate the analysis pipeline described herein.

27. Sequencing quality control.
  a. Perform quality control on raw FASTQ files using FastQC:
    > fastqc sample_R1.fastq.gz sample_R2.fastq.gz -o fastqc_reports/
  b. If adapter contamination or low-quality bases are detected, trim reads using Trimmomatic:
    > trimmomatic PE sample_R1.fastq.gz sample_R2.fastq.gz \
    sample_R1_trimmed.fastq.gz sample_R1_unpaired.fastq.gz \
    sample_R2_trimmed.fastq.gz sample_R2_unpaired.fastq.gz \
    ILLUMINACLIP:adapters.fa:2:30:10 LEADING:3 TRAILING:3 \
    SLIDINGWINDOW:4:15 MINLEN:36
  c. After trimming, re-run FastQC to confirm minimal adapter contamination.
    ***Note:*** Per-base quality scores should predominantly exceed Q30 (≥90–95% of bases). Check for GC content, duplication rate, and overrepresented sequences (adapter contamination).
28. Read mapping.
  a. Align trimmed reads to the appropriate reference genome (e.g., mm10 for mouse) using Bowtie2^12^:
    > bowtie2 -x mm10_index -1 sample_R1_trimmed.fastq.gz -2
    sample_R2_trimmed.fastq.gz \
    --very-sensitive -p 8 2> sample_align.log | samtools
    view -bS - >
  b. Sort and index the BAM file using SAMtools:
    > samtools sort sample.bam -o sample_sorted.bam
    > samtools index sample_sorted.bam
  c. Generate alignment statistics to assess mapping quality:
    > samtools flagstat sample_sorted.bam > sample_flagstat.txt
29. Cross-correlation analysis.
  a. Evaluate the quality of PD-seq data by assessing the cross-correlation between reads on plus and minus strands. Compute cross-correlation using phantompeakqualtools (run_spp.R):
    > Rscript run_spp.R -c=sample_sorted.bam -savp
    -out=sample.cc.qc.txt
  b. Extract key QC metrics from the output:
    i. NSC (Normalized Strand Cross-correlation coefficient): ratio of cross-correlation at fragment length to background.
    ii. RSC (Relative Strand Cross-correlation coefficient): ratio of cross-correlation at fragment length to cross-correlation at read length.
    iii. Estimated fragment length.
  c. Expected ranges (ENCODE-style practical thresholds):
    i. NSC > 1.05: acceptable; > 1.10: good.
    ii. RSC > 0.8: acceptable; > 1.0: good.
    iii. RSC < 0.5: poor enrichment or high background.
      ***Note:*** For true binding events, reads should be offset by approximately the fragment length. These are guidelines, not absolute rules. PD-seq may behave differently from canonical TF ChIP-seq; therefore, replicate concordance and downstream peak quality should also be considered.
30. Peak calling with MACS2.
  a. Call peaks using MACS2^13^:
    > macs2 callpeak -t FoxP3_sorted.bam \
    -c Input_sorted.bam/MBP_sorted.bam \
    -f BAMPE -g mm -n FoxP3_PDseq \
    --outdir macs2_output/ -q 0.05
  b. Visualize peaks in IGV (Integrative Genomics Viewer) to confirm enrichment patterns at expected regions.
    ***Note:*** Use both Input and MBP samples as controls to ensure high-confidence results. Input corrects for sequence-specific biases, while MBP excludes non-specific binding to the affinity tag.
31. FRiP (Fraction of Reads in Peaks) Calculation.
  a. Calculate FRiP using BEDTools and SAMtools (FRiP = reads in peaks/total mapped reads):
    > cut -f1-3 FoxP3_PDseq_peaks.narrowPeak > FoxP3_peaks.bed
    > samtools view -b -L FoxP3_peaks.bed
    FoxP3_sorted.bam > peaks_reads.bam
    > samtools flagstat FoxP3_sorted.bam > total_stats.txt
    > samtools flagstat peaks_reads.bam > peak_stats.txt
  b. Interpret FRiP values:
    i. <0.01: very weak enrichment.
    ii. 0.01–0.05: modest enrichment.
    iii. 0.05–0.20: good enrichment.
    iv. >0.20: strong enrichment.
      ***Note:*** FRiP measures the proportion of total reads that overlap with called peaks, providing an indication of signal enrichment.
32. Motif analysis.
  a. Extract DNA sequences from peak regions (e.g., 200 bp centered on peak summits):
    > bedtools slop -i FoxP3_PDseq_summits.bed -g
    mm10.chrom.sizes -b 100 > summits_200bp.bed
    > bedtools getfasta -fi mm10.fa -bed
    summits_200bp.bed -fo FoxP3_peaks.fa
  b. Perform de novo motif discovery using HOMER or MEME-ChIP.^14^
    i. HOMER example:
      > findMotifsGenome.pl FoxP3_PDseq_peaks.narrowPeak
      mm10 FoxP3_motifs/ -size 200 -len 8,10,12,15,20
    ii. MEME-ChIP example:
      > meme-chip -oc meme_output/ -meme-mod anr -meme-minw 6 -meme-maxw 30 \ -dreme-e 0.05 -centrimo-maxreg 200 FoxP3_peaks.fa
  c. Cross-reference with controls: Compare motif enrichment between FoxP3 pulldown samples and Input/MBP controls to ensure specificity. troubleshooting 5.
    In the case of FOXP3, this analysis robustly reveals a T_n_G microsatellite repeat (e.g., T3G) as the predominant motif, rather than the canonical FKHM (TGTTTAC) (Figure 7C). Subsequent experimental validation further confirmed that this newly identified FOXP3-binding pattern plays a major role in regulating Treg functionality.^15^^,^^16^ troubleshooting 5.

## Expected outcomes

Faithful execution of the PD-seq protocol will generate high-quality biochemical and sequencing data that define the intrinsic DNA-binding specificity of the TF of interest. The following summarizes the expected results for each stage of the workflow, using FOXP3 as an illustrative model.

Pull-down with synthetic DNA oligos serves as a functional quality control for the purified protein. SYBR Gold-stained native gels should show a strong band for the high-affinity positive-control oligo (e.g., IR-FKHM), a moderate band for the canonical FKHM motif, and no detectable band for the negative-control oligo lacking FOXP3-binding sites. These differential enrichment patterns confirm protein integrity and sequence-specific binding activity. Relative band intensities may be quantified using ImageJ.

Recombinant protein induction and purification should yield >5 mg/L of soluble protein such as His-MBP-FOXP3(ΔN). SDS-PAGE analysis should reveal a single dominant band at the expected molecular weight (e.g., ∼70 kDa) with >90% purity, and heparin chromatography should produce a sharp, symmetric elution peak indicative of a homogeneous preparation. Protein identity may be validated by anti-MBP western blotting, and concentration determined by Bradford assay.

Genomic DNA extraction should produce high-quality naked genomic DNA with an A260/A280 ratio of ∼1.8. Following controlled DNase I digestion, the fragmented DNA should migrate as a smooth smear centered at ∼50-200 bp on a 2% agarose gel or display a single Bioanalyzer peak within this range. This fragment size ensures adequate context for de novo motif discovery while maintaining binding-site resolution.

Following incubation and washing, the recovered DNA-typically in the nanogram range-should be significantly higher in the MBP-TF pulldown than in the MBP-only control. Quantification using a high-sensitivity fluorometric assay (e.g., Qubit) is recommended. Optional qPCR analysis against known positive and negative genomic loci can provide rapid assessment of enrichment specificity prior to library construction.

Properly constructed libraries should show a dominant Bioanalyzer peak at 170–290 bp, reflecting the insert plus ∼120 bp of Illumina adapters. Adapter dimer contamination (∼120 bp) should be minimal. Sequencing on an Illumina platform with 2×38 bp or 2×50 bp read lengths typically yields 20-50 million high-quality reads per sample, which is sufficient for robust peak calling.

Peak calling (e.g., using MACS2) should reveal genomic regions enriched specifically in the MBP-TF sample relative to controls. De novo motif analysis (e.g., MEME-ChIP, HOMER) is expected to identify one or more statistically significant intrinsic binding motifs. For FOXP3, PD-seq robustly detects a T_n_G microsatellite repeat (e.g., T_3_G) as the top motif, which shows far stronger enrichment than the canonical FKHM (TGTTTAC). Synthesizing and validating this motif in vitro should confirm its high binding affinity. These results highlight PD-seq’s ability to uncover intrinsic TF-binding patterns often masked in chromatin-based assays.

## Limitations

PD-seq and ChIP-seq are not interchangeable but complementary techniques. PD-seq measures the intrinsic DNA-binding specificity of a TF in a purified biochemical system, reflecting the sequences that a TF is capable of binding in isolation. In contrast, ChIP-seq reveals the actual genomic occupancy of the TF under specific cellular conditions, within the context of chromatin, co-factors, and other regulatory influences. Therefore, the binding motifs identified by the two methods may differ. To fully understand the biological function of a TF, it is advisable to integrate both datasets and to perform biological validation-such as through ATAC-seq, ChIP-based methods, or functional assays-in order to evaluate their physiological relevance.

High TF concentrations and naked DNA can enrich weak or low-affinity binding events that would be inaccessible in vivo. Careful optimization of protein: DNA ratios and inclusion of appropriate controls (e.g., affinity tag-only conditions) help minimize false positives.

PD-seq identifies sequences a TF can bind, but not the consequences of such binding in specific cell types or regulatory contexts. Additional experiments are required to establish effects on transcriptional activation, repression, or chromatin remodeling.

## Troubleshooting

### Problem 1

DNA Fragments Are Too Short or Long (related to step 17).

### Potential solution

Pre-calibrate digestion parameters by performing a time-course or concentration-gradient assay using a small aliquot of genomic DNA (e.g., 5 μg). Analyze fragment sizes via agarose gel electrophoresis or Bioanalyzer after 1, 2, 3, 4, and 5 minutes to identify optimal conditions yielding 50-200 bp fragments.

### Problem 2

Low DNA Yield After Pull-Down and Elution (related to step 21).

### Potential solution

Scale up the reaction system to increase input DNA and protein quantities. Optimize binding buffer components (e.g., adjust salt concentration) to enhance stability and specificity. Ensure the starting DNA amount meets the minimum requirement for library construction.

### Problem 3

High Background in MBP-Only Control (related to step 23).

### Potential solution

Increase wash stringency by (1) raising the number of washes (e.g., from 3 to 5), (2) incorporating mild detergents (e.g., 0.1% Triton X-100) into wash buffers, and (3) adding non-specific competitor DNA (e.g., 100 μg/mL sonicated salmon sperm DNA or poly-dIdC) to block non-specific sites during binding and washing.

### Problem 4

No or Weak Peaks Detected After Sequencing (related to step 26).

### Potential solution

Ensure sequencing depth reaches 20–50 million reads per sample. Perform pre-sequencing quality controls (e.g., qPCR and Bioanalyzer) to confirm library purity, concentration, and absence of adapter dimers.

### Problem 5

Expected Motifs Not Enriched in Sequencing Results (related to step 32).

### Potential solution

Embrace de novo motif discovery to identify non-canonical binding sites (e.g., T_n_G repeats for FOXP3). Validate findings by synthesizing candidate oligonucleotides and performing in vitro binding assays. Follow up with physiological or structural studies to confirm biological relevance.

## Resource availability

### Lead contact

Further information and requests for resources and reagents should be directed to the lead contact, Dr. Wenxiang Zhang (wenxiang.zhang@sjtu.edu.cn).

### Technical contact

Questions about the technical specifics of performing the protocol should be directed to and will be fulfilled by the technical contact, Dr. Wenxiang Zhang (wenxiang.zhang@sjtu.edu.cn) and Ms. Xuan Jiang (jiangxuan629@sjtu.edu.cn).

### Materials availability

The plasmid His-MBP-FOXP3(ΔN) and related constructs were generated as previously described.^1^ No unique reagents were generated that require a materials transfer agreement beyond standard institutional procedures.

### Data and code availability

The naked genomic DNA PD-seq data generated in this protocol have been deposited in the Gene Expression Omnibus (GEO) under accession number GEO: GSE243606 and are publicly available.

## Acknowledgments

We thank Prof. Sun Hur (10.13039/100006691Harvard Medical School) for insightful guidance and conceptual support during the development of this methodology. This work was supported by the 10.13039/100016698National Natural Science Foundation of China (32571039), the Shanghai Pujiang Talent Program (Category A) (24PJA111), and the 10.13039/100017950Shanghai Municipal Health Commission Medical New Technology and Transformation Seed Program (2024ZZ2013).

## Author contributions

W.Z. conceived the project, designed the methodology, and supervised all aspects of protocol development. W.Z. developed and optimized the PD-seq workflow. W.Z. and X.J. performed the majority of the experiments, including protein purification, genomic DNA preparation, pull-down assays, and library construction. W.Z. and X.J. analyzed the data and prepared the figures. W.Z. and X.J. co-wrote the manuscript. All authors reviewed and approved the final version of the protocol.

## Declaration of interests

The authors declare no competing interests.
